# Different Cell Wall-Degradation Ability Leads to Tissue-Specificity between *Xanthomonas oryzae* pv*. oryzae* and *Xanthomonas oryzae* pv*. oryzicola*

**DOI:** 10.3390/pathogens9030187

**Published:** 2020-03-04

**Authors:** Jianbo Cao, Chuanliang Chu, Meng Zhang, Limin He, Lihong Qin, Xianghua Li, Meng Yuan

**Affiliations:** 1 National Key Laboratory of Crop Genetic Improvement, National Center of Plant Gene Research (Wuhan), Huazhong Agricultural University, Wuhan 430070, China; clchu@webmail.hzau.edu.cn (C.C.); mengzhang@webmail.hzau.edu.cn (M.Z.); xhli@mail.hzau.edu.cn (X.L.); 2 Public Laboratory of Electron Microscopy, Huazhong Agricultural University, Wuhan 430070, China; liminhe@whut.edu.cn (L.H.); qlh@mail.hzau.edu.cn (L.Q.)

**Keywords:** *Xanthomonas oryzae* pv. *oryzae*, *Xanthomonas oryzae* pv. *oryzicola*, rice, cell wall-degradation, tissue-specificity

## Abstract

*Xanthomonas oryzae* pv. *oryzae* (*Xoo*) and *Xanthomonas oryzae* pv. *oryzicola* (*Xoc*) lead to the devastating rice bacterial diseases and have a very close genetic relationship. There are tissue-specificity differences between *Xoo* and *Xoc*, i.e., *Xoo* only proliferating in xylem vessels and *Xoc* spreading in intercellular space of mesophyll cell. But there is little known about the determinants of tissue-specificity between *Xoo* and *Xoc*. Here we show that *Xoc* can spread in the intercellular spaces of mesophyll cells to form streak lesions. But *Xoo* is restricted to growth in the intercellular spaces of mesophyll cells on the inoculation sites. In vivo, *Xoc* largely breaks the surface and inner structures of cell wall in mesophyll cells in comparison with *Xoo*. In vitro, *Xoc* strongly damages the cellulose filter paper in comparison with *Xoo*. These results suggest that the stronger cell wall-degradation ability of *Xoc* than that of *Xoo* may be directly determining the tissue-specificity.

## 1. Introduction

*Xanthomonas oryzae* pv. *oryzae* (*Xoo*) and *Xanthomonas oryzae* pv. *oryzicola* (*Xoc*) lead to bacterial blight and bacterial leaf streak in rice (*Oryza sativa* L.), respectively [1]. Bacterial blight and bacterial leaf streak are devastating bacterial disease of rice worldwide [1,2]. *Xoo* and *Xoc* both belong to *Xanthomonas* species in the Gammaproteobacteria and have a very close genetic relationship [1,2]. However, *Xoo* enters rice leaf through hydathodes or wounds then multiplies in xylem vessels to cause leaf blight [1]. In contrast, *Xoc* gains access to leaf by stomata or wounds and multiplies in the sub-stomatal cavity [1]. *Xoc* can spread in the intercellular spaces of mesophyll cells to form streak lesions between the veins [1]. But the determinants of *Xoo* and *Xoc* parasitic tissue-specificity are still largely unknown [3]. 

Many factors possibly determine the tissue-specificity of *Xanthomonas* species among vascular and non-vascular pathogens. The minimal distinguishing differences, such as the subtler difference of amino acid and non-coding nucleotide polymorphisms, maybe determine the tissue-specificity between vascular and non-vascular pathogens [4]. The four positions of amino acid residue from HpaA and XpsD proteins correlate with tissue-specificity among *Xoc* strain BLS256, *Xoo* strain KACC100331 and MAFF311018, and other vascular or non-vascular *Xanthomonas* strains [5]. HpaA is a secreted and translocated protein as the type III secretion system (T3SS) components that affect the secretion of effectors and translocators [5,6]. XpsD is an outer membrane protein that serves as a gatekeeper for the type II secretion system (T2SS) and directs the secretion of different extracellular proteins in species for functioning in specific tissues [5,7]. 

T2SS and T3SS play important roles in the pathogenesis and the virulence factor secretion of *Xoo* and *Xoc*. The T2SS in *Xanthomonas* consists of at least 12 membrane proteins on the outer membrane of the bacterial cell [8]. T2SS is responsible for the extracellular secretion of toxins, proteases, cellulases and lipases, which are required for virulence and pathogenesis in *Xanthomonas* [8,9]. Mutations in genes of the T2SS components, cAMP regulatory protein and extracellular protease A of *Xoc* strain RS105 can impair the extracellular protease activity and reduce the virulence of *Xoc* bacteria in rice [8]. Furthermore, the *ecpA_Xoo_* from PXO99^A^ with a frame shift, which is also different from the EcpA*_Xoc_* of RS105 in C-terminal residues, may cause the loss of extracellular protease A (EcpA) activity [8]. It is suggested that the EcpA*_Xoc_* is a tissue-specific virulence factor for *Xoc* [8]. The T3SS is responsible for the delivery of transcription activator-like (TAL) effectors into plant cell nuclei [10]. Many TAL effectors of *Xoo* activate the transcript of rice susceptibility or resistant genes [2,11]. However, except for Tal2a effector eliciting dose-dependent resistance, most TAL effectors of *Xoc* suppress the innate immunity response of rice against *Xoc* [11]. It is implied that the TAL effectors secreted by T3SS maybe determinate the tissue-specificity between *Xoo* and *Xoc*.

The *feoABC* (Ferrous iron transporter) system may regulate *Xoo* tissue-specific adaption to rice xylem vessels according to the following evidences [12]. Firstly, the *feo* genes of *Xoo* are only induced by xylem tissues of infected rice and no expression of *xss* (*Xanthomonas* siderophore synthesis) operon could be detected in xylem tissues [13]. Secondly, the *xsu*-xss (*Xanthomonas* siderophore utilization) operon of *Xoc* is expressed during *Xoc* infecting the rice mesophyll tissue [14]. Thirdly, the xylem vessels contain enough Fe^3+^-citrate complex which is sufficient for bacterial growth but mesophyll tissue may have little Fe^2+^ or Fe^3+^ irons [15].

The cell wall-degradation enzymes (CWDEs) such as lipase, cellulase, xylanase, and endoglucanase secreted by the T2SS of *Xoo* contribute to disease symptoms [8,16]. There are few reports about the function of CWDEs secreted by *Xoc* [8]. Only the *ecpA* mutant of *Xoc* can cause no xylanase or cellulase activities of bacteria and is avirulent to rice [8]. Whether CWDEs are correlated with the tissue-specificity of *Xoo* and *Xoc* should be analyzed.

To further investigate the direct determinants of tissue-specificity between *Xoo* and *Xoc*, we compared the structural differences of cell walls, which are in direct contact with *Xoo* and *Xoc* by infiltration inoculation methods [17,18], in mesophyll cells of susceptible rice lines. We found that the cell wall-degradation ability of *Xoc* was stronger than that of *Xoo* in in vivo and in vitro conditions. These findings imply that the difference of cell wall-degradation ability in *Xoo* and *Xoc* contributes to the tissue-specificity between *Xoo* and *Xoc*.

## 2. Materials and Methods

### 2.1. Bacterial Strains and Rice Cultivar

*Xoo* strain PXO99^A^ and *Xoc* strain RH3 were obtained from China General Microbiological Culture Collection Center and International Rice Research Institute [18,19]. The susceptible cultivar IR24 belongs to the *indica* (*Oryza sativa* ssp. *indica*) subgroup of Asian cultivated rice [18]. 

### 2.2. Pathogen Inoculation and Filter Paper in Vitro Assay

Rice plants at 4-leaf stage were inoculated by infiltrating leaves with 10^9^ cells ml^-1^ of *Xoo* and *Xoc* bacterial suspension by using a needleless syringe [17]. The sterile cellulose filter paper strips (2 mm × 3 cm) embedded in *Xoo* and *Xoc* bacterial exudes on potato dextrose agar (PDA) for 5 days. At the 5^th^ day, the filter paper strips were observed by using normal field-emission scanning electron microscope. All the inoculation of plants and filter paper treatment with *Xoo* and *Xoc* were biologically carried out at least twice with similar results, and one replicate was shown.

### 2.3. Normal and Cryo Field-Emission Scanning Electron Microscopy

On the corresponding day, the rice leaves at the inoculation sites were longitudinally cut for exposing cell wall and were then cut into 2 mm × 2 mm blocks with new razors and the filter paper strips were cut into 2 mm × 2 mm blocks, then the leaf blocks and the small strips were immediately immersed in 2.5% (v/v) glutaraldehyde in sodium phosphate buffer (0.1 M, pH 7.2) at 4 ℃ for 12 h. The sample blocks were dehydrated in a gradient series of ethanol, then dried in the critical point equipment (HCP-2, Hitachi, Tokyo, Japan), sputter-coated with platinum in sputtering apparatus (MCIOO, Hitachi, Tokyo, Japan), and observed with a field-emission scanning electron microscope (SU8010, Hitachi, Tokyo, Japan). To quantify the images of cell wall with cellulose microfibrils at high magnification and the images with interstitial cavity between fibers or cellulose microfibrils on filter paper, 8–14 images were obtained from three plants in two independent inoculations and 4–15 images were obtained from three filter papers in one treatment.

For in vivo analyzing the growth of bacteria on rice leaf, the fresh rice leaves with inoculation sites and non-inoculation sites were rapidly frozen in solid liquid-nitrogen, sublimated, and sputter-coated with platinum in cryo-scanning electron microscopy transfer system (PP3010T, Quorum, London, England). The coated leaves were observed under a field-emission scanning electron microscope (SU8010, Hitachi, Tokyo, Japan) at 1 kV and a working distance of 8.3 mm.

### 2.4. Transmission Electron Microscopy

The leaf tissues at the inoculation site and non-inoculation site (adjacent to inoculation site) were cut into 2 mm × 1 mm blocks which were then fixed in 2.5% (v/v) glutaraldehyde in sodium phosphate buffer (0.1 M, pH 7.2) at 4 ℃ overnight, and washed for 30 min with the same buffer three times. The leaf blocks were post-fixed in 1% (w/v) osmium tetroxide for 2 h, washed 30 min with the same buffer three times, and dehydrated in a series of acetone concentration. Dehydrated blocks were progressively infiltrated and embedded in Spurr’s resin (SPI, SPI Chem, West Chester, United States), then polymerized at 65 ℃ for 48 h. The samples were cut into ultrathin sections (60–70 nm thick) with diamond knife, stained with 2% uranyl acetate, and observed with a Hitachi transmission electron microscope (H-7650, Hitachi, Japan) at 80 kV.

### 2.5. Cellulose Synthase Gene Expression Analysis

Total RNA of rice leaves isolated from the 3-cm leaf fragments including inoculations sites, was extracted by using Trizol reagent (Invitrogen, Carlsbad, CA, USA). An aliquot (5 µg) of total RNA was firstly treated with RNase-free DNase I (Invitrogen, Carlsbad, CA, USA) to remove potentially contaminating DNA, and then first-strand cDNA was reverse transcribed with oligo(dT)_18_ primer using M-MLV reverse transcriptase (Promega, Madison, WI, USA) according to the manufacturer’s protocols. Quantitative reverse transcription polymerase chain reaction (qRT-PCR) was carried out by using cellulose synthase gene (*CESA*) specific primers (Supplementary Table S1), as described previously [20]. The transcript level of rice actin gene was used to standardize the RNA sample of each RT-PCR, and the expression level relative to that of IR24 inoculated by PXO99^A^ at 0 h was calculated. Each qRT-PCR assay was biologically carried out twice with similar results, within each repetition having three technical replicates and only one biological replicate was presented.

### 2.6. Statistical Analysis

The significant differences among lesion length, percentage of images with cellulose microfibril, percentage of image with interstitial cavity between fibers or cellulose microfibrils on filter paper and relative expression level of *CESA* genes were assessed using pairwise Student’s *t*-test in Excel (Microsoft, http://www.microsoftstore.com).

## 3. Results

### 3.1. The Leaf Tissue Morphology of Susceptible Rice Infected with Xoo and Xoc

**Figure 1:**
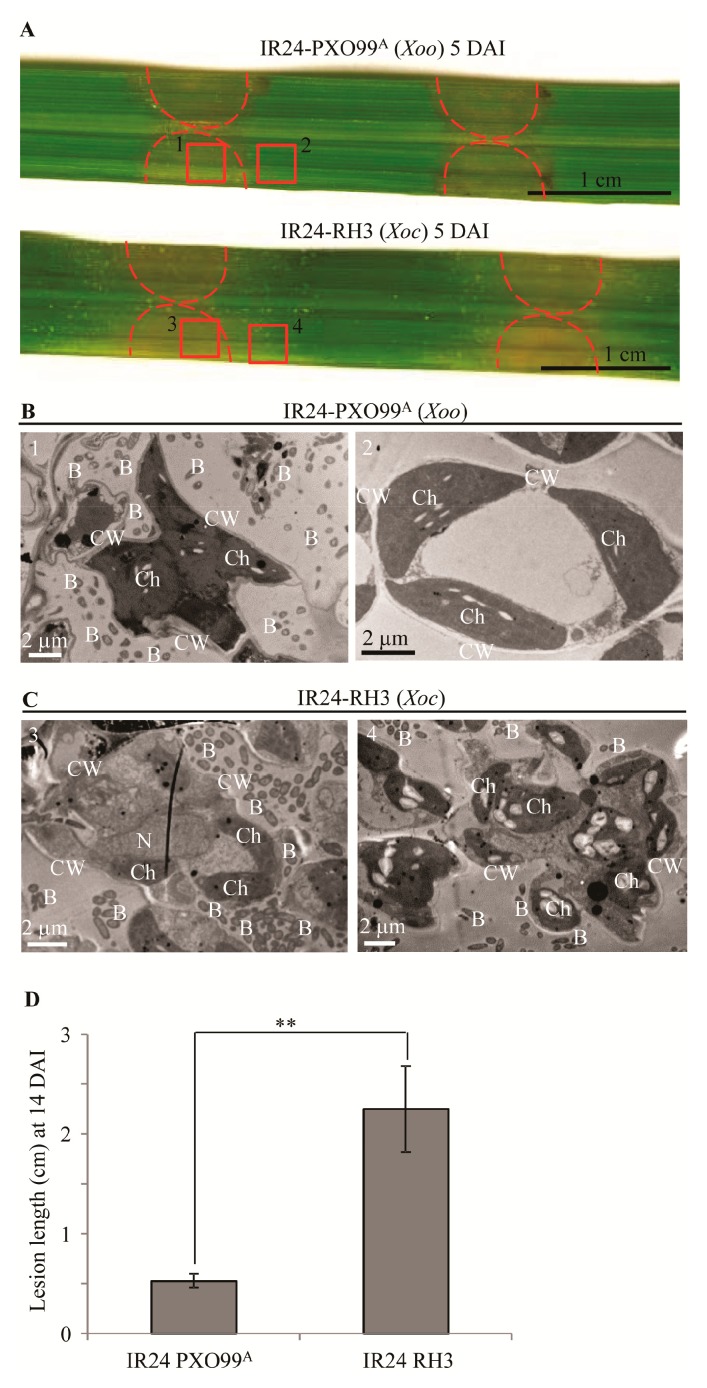
*Xoo* only multiplied around mesophyll cells at inoculation site, but *Xoc* spread through mesophyll cells. DAI, days after inoculation; B, *Xoo* or *Xoc* bacterium; Ch, Chloroplast; CW, cell wall; N, nucleus; The inside area of dotted red arc, the area of inoculation site; Red square frame 1 and 3, inoculation site; Red square frame 2 and 4, non-inoculation site. (**A**) Leaf response of susceptible rice IR24 inoculated *Xoo* strain PXO99^A^ and *Xoc* strain RH3. (**B**,**C**) Ultrastructural feature of mesophyll cells in inoculation sites (1,3) and non-inoculation sites (2,4). (**D**) Lesion length of IR24 infected with *Xoo* strain PXO99^A^ and *Xoc* RH3 at 14 DAI. Bar represent mean (5 to 12 leaves from three plants) ± standard deviation (SD). Double asterisks (**) stand for the significant difference between IR24 inoculated with RH3 and IR24 inoculated with PXO99^A^ at *p* < 0.01.

**Figure 2:**
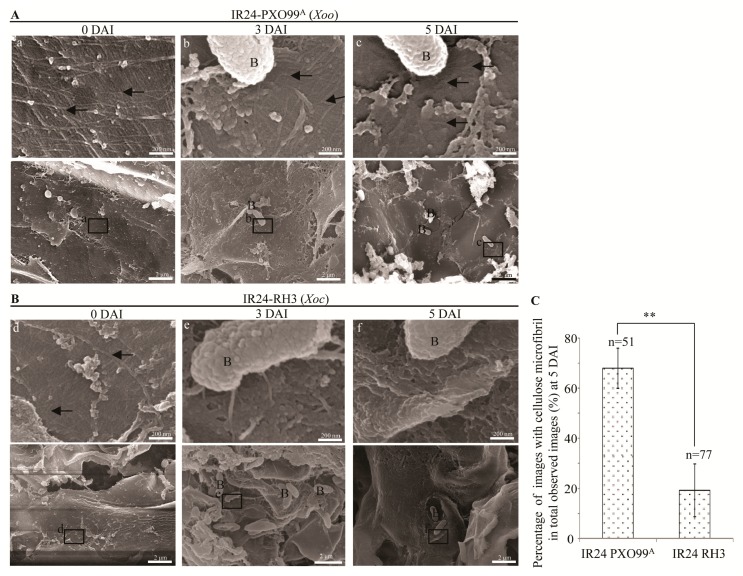
The intact cell wall of mesophyll cells in rice infected with *Xoo*, but the broken cell wall in rice infected with *Xoc*. B, bacterium; black arrow, cellulose microfibril. (**A**) The intact surface structures of cell wall in mesophyll cells of rice leaves inoculated with *Xoo* strain PXO99^A^ represented by low magnification images (down) and high magnification images (upper) at 0, 3, and 5 day after inoculation (DAI). (**B**) The broken surface structures of cell wall in mesophyll cells of rice leaves infected with *Xoc* strain RH3 at 3 and 5 DAI in comparison with plants at 0 DAI represented by low magnification images (down) and high magnification images (upper). (**C**) The percentage of images with cellulose microfibril in total observed high magnification images from six different plants at 5 DAI. Data represent mean (at least six mesophyll cells were observed from six different plants in two independent inoculation) ± standard deviation (SD). Double asterisks (**) stand for the significant difference between percentage of image with cellulose microfibril in rice inoculated with PXO99^A^ and percentage of image with cellulose microfibril in rice inoculated with RH3 at *p* < 0.01. n, the total number of observed high magnification images.

**Figure 3:**
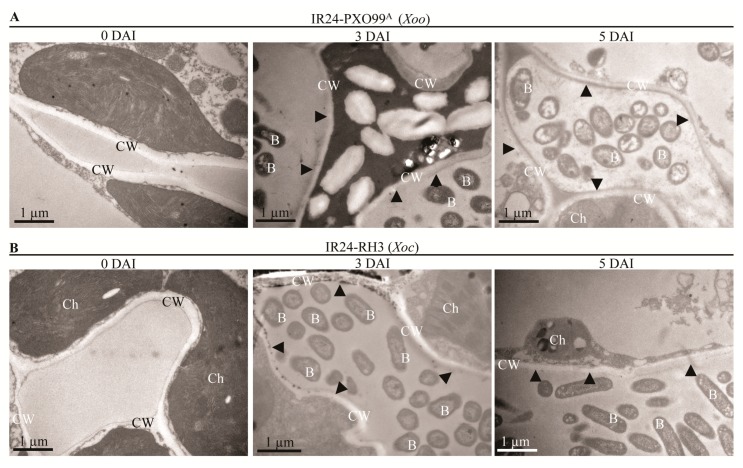
The lower electron-density of cell wall degraded by *Xoo* in comparison with the cell wall degraded by *Xoc*. CW, cell wall; B, *Xoo* or *Xoc* bacterium; Ch, chloroplast; black arrow head, the cell wall of mesophyll. (**A**) The normal electron-density layers of cell wall in mesophyll cells of rice IR24 leaves inoculated with *Xoo* strain PXO99^A^ at 3, 5 DAI in comparison with cell wall of rice leaves at 0 DAI. (**B**) The higher electron-density layers of cell wall with electron-dense particles in mesophyll cells of rice IR24 leaves inoculated with *Xoc* strain RH3 at 3, 5 DAI in comparison with cell wall of rice leaves at 0 DAI.

**Figure 4:**
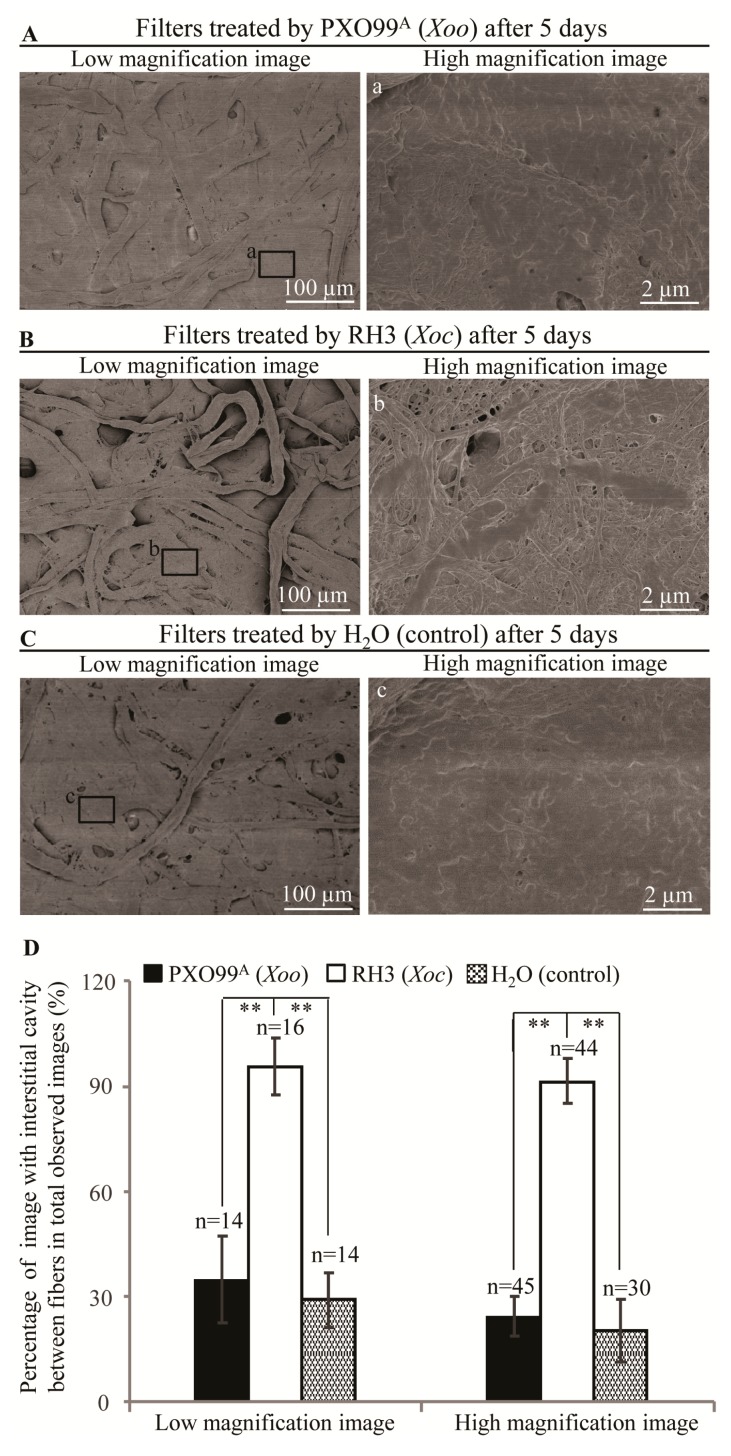
In vitro lower degradation ability of *Xoo* than that of *Xoc*. (**A**) The fibers densely packing and few interstitial cavities between microfibrils on surface of filter paper treated by *Xoo* strain PXO99^A^ at the 5^th^ day. (**B**) Many interstitial cavities between big fibers and microfibrils on surface of filter paper treated by *Xoc* strain RH3 at the 5^th^ day. (**C**) The fibers densely packing and few interstitial cavities between microfibrils on surface of filter paper treated by H_2_O (negative control) at the 5^th^ day. (**D**) The percentage of image with interstitial cavity between fibers in total observed images from filter papers treated by *Xoo* strain PXO99^A^, *Xoc* strain RH3 and H_2_O at the 5^th^ day. Data represent mean (at least three images were observed from three different filter papers) ± standard deviation (SD). Double asterisk (**) stand for the significant differences between percentage of images with interstitial cavities among fibers or microfibrils between infilter paper treated by PXO99^A^ and infilter paper treated by RH3, between infilter paper treated by RH3 and infilter paper treated by H_2_O (negative control) at *p* < 0.01. n, the total number of observed images.

Leaf is a major infection site of *Xoo* and *Xoc* [1]. To study whether *Xoo* can grow and multiply in the extracellular spaces of mesophyll cells, we firstly analyzed the leaf phenotypes of susceptible rice IR24 inoculated with *Xoo* strain PXO99^A^ and *Xoc* strain RH3. On the inoculation sites, the leaves inoculated with *Xoo* represented water-soaked symptom with little brown color around inoculation sites and the leaves inoculated with *Xoc* were yellow colored at 5 days after infection (DAI) (Figure 1A). There were many bacteria around mesophyll cells in leaf inoculated by *Xoo* and *Xoc* on inoculation sites (Figure 1B-1,C-3). In the non-inoculation sites (adjacent to the inoculation site), only rice leaves inoculated with *Xoc* represented yellow streak lesions with yellow exudates on leaf surface (Figure 1A) and contained many bacteria around the mesophyll cells (Figure 1C-4); but there was no bacterium around the mesophyll cells in rice leaf inoculated by *Xoo* (Figure 1B-2). At 5 DAI, some bacterial exudates showed on stoma of inoculation site for both leaves inoculated with *Xoo* and *Xoc* (Supplementary Figure S1); but there were many bacterial exudates on stoma of non-inoculation site (adjacent to the inoculation site) leaf inoculated with *Xoc* and no bacterial exudates on stoma of non-inoculation site leaf inoculated with *Xoo* (Supplementary Figure S1). At 14 DAI, the lesion length of the rice leaf inoculated by *Xoo* strain PXO99^A^ was only 0.5 cm (the diameter of inoculation syringe was 0.5 cm); the lesion length of rice leaf inoculated by *Xoc* strain RH3 exceeded 2 cm (Figure 1D).

### 3.2. The Cell Wall Surface Structures of Mesophyll Cells in Rice Infected with Xoo and Xoc 

Plant cell wall is the first physical barrier against pathogens in plant-pathogen interaction [21]. Therefore, we analyzed the cell wall structures of susceptible rice IR24 leaves infected with *Xoo* and *Xoc*. In leaves infected with *Xoo* strain PXO99^A^, the cell wall surface of the mesophyll cells was still flat (low magnification images) and represented many cellulose microfibrils (black arrow) (high magnification images) at 3 and 5 DAI which were almost similar to a lot of cellulose microfibrils on the flat surface of cell wall at 0 DAI (Figure 2A). In rice leaves infected with *Xoc* strain RH3, the cell wall surface of mesophyll cells had more pits (low magnification images), especially on the sites of cell wall in contact with bacterium, and fewer cellulose microfibrils (high magnificent images) at 3 and 5 DAI in comparison with the flat cell wall surface and many cellulose microfibrils at 0 DAI (Figure 2B). At 5 DAI, the number of images with cellulose microfibrils (high magnification image) from leaves inoculated with *Xoo* strain PXO99^A^ were significantly (*p* < 0.01) more than the number of images with cellulose microfibrils from leaves inoculated with *Xoc* strain RH3 (Figure 2C).

### 3.3. The Cell Wall Inner Structure of Mesophyll Cells in Rice Infected with Xoo and Xoc

Under transmission electron microscopy (TEM) analyzing rice mesophyll cells, the normal cell wall represented low electron-density (transparent) layer structure; the cell wall broken by pathogens represented high electron-density (opaque) layer structure because of osmiophilic material deposition in cell wall [22,23]. To further investigate the cell wall inner structure, we observed the cell wall of the mesophyll cell in susceptible rice IR24 inoculated by *Xoo* strain PXO99^A^ and *Xoc* strain RH3 under TEM. There was slightly higher electron density of cell wall (shown by black arrow head) and distinct layers of cell wall in mesophyll cells of leaves inoculated with *Xoo* at 3, 5 DAI in comparison with the electron-density of cell wall at 0 DAI (Figure 3A). However, there was dramatically higher electron-density of cell wall (black arrow head) and non-layers of cell wall in mesophyll cells of leaves inoculated with *Xoc* at 3, 5 DAI in comparison with the electron density of cell wall at 0 DAI (Figure 3B). At the same time, the out layer of the cell wall contacting with the bacterium consisted of many electron-dense (osmiophilic) particles in mesophyll cell of leaves inoculated with *Xoc* at 3 DAI (Figure 3B). Cellulose as the major component of plant cell wall is synthesized by cellulose synthase genes (*CESA*) [21]. To further analyze the cell wall inner structure, we found that the relative expression levels of *CESA4*/*7*/*9* significantly reduced in IR24 rice leaves inoculated both with *Xoo* strain PXO99^A^ and with *Xoc* strain RH3 (Supplementary Figure S2). However, the expression of *CESA4*/*7*/*9* in rice leaves inoculated by *Xoc* were significantly (*p* < 0.01 and *p* < 0.05) induced to lower levels than in rice leaves inoculated by *Xoo* at 2, 4, 12, and 24 hours after inoculation (Supplementary Figure S2).

### 3.4. The Cellulose Filter Papers Less Damaged by Xoo than Xoc

The qualitive filter paper (Grade 1, GE Whatman, Hangzhou, China), which mostly consisted of cellulose, hemicellulose, and lignin (www.gelifesciences.com), were treated with bacteria to in vitro analyze the cell wall-degradation ability of *Xoo* strain PXO99^A^ and *Xoc* strain RH3. After 5 days, the surface of filter paper treated by *Xoo* showed few interstitial cavities between big fibers in low magnification image and no interstitial cavities between the densely packing microfibrils in high magnification image (Figure 4A); however, the surface of filter paper treated by *Xoc* showed many interstitial cavities between big fibers in low magnification image and many interstitial cavities between microfibrils in high magnification image (Figure 4B). Meanwhile, the surface of filter paper treated by H_2_O (negative control) also showed few interstitial cavities between big fibers or microfibrils after 5 days treatment (Figure 4C). The number of images with interstitial cavities between fibers or microfibrils in filter paper treated by *Xoc* strain RH3 was 3-fold higher than the number of images with interstitial cavities between fibers or microfibrils in filter paper treated by *Xoo* strain PXO99^A^ or H_2_O (Figure 4D).

## 4. Discussion

In rice leaf tissue, *Xoo* proliferates in xylem vessel, while *Xoc* multiplies in intercellular spaces of mesophyll tissue [1]. However, many wild type *Xoo* strains and *Xoc* strains, which are infiltrated into mesophyll tissue after 3–7 days, can both cause water-soaked lesions on leaves of susceptible rice varieties [17,24,25]. At the 5^th^ day after infiltration inoculation, *Xoo* and *Xoc* proliferated in the intercellular space between mesophyll cells, which resulted in yellow lesions and bacterial exudate formations on inoculation sites of leaves (Figure 1B-1,C-3; Supplementary Figure S1). These evidences suggest that *Xoo* and *Xoc* can both grow in intercellular spaces between mesophyll cells. But at the non-inoculation sites (adjacent to inoculation site) of leaves inoculated by *Xoo*, no *Xoo* bacteria were observed in the intercellular spaces between mesophyll cells following by no lesions and bacterial exudate formations (Figure 1A,B-2; Supplementary Figure S1A). However, many bacteria of *Xoc* were growing in the intercellular spaces between mesophyll cells which caused streak lesions and bacterial exudates formations on the leaves of non-inoculation sites (Figure 1A,C-4; Supplementary Figure S1B). Almost all the wild *Xoc* strains can infect *indica* and *japonica* rice leaves to form streak lesions along the veins [1,2,8,11]. The mesophyll tissue of rice leaf is composed of the lobed mesophyll cells that are joined to one another by the tight fusion cell wall of the lobes [26]. So, *Xoc* bacteria must break the cell wall junction between the lobes of mesophyll cell then spread to the neighbor intercellular spaces between mesophyll cells in rice leaf. These evidences indicate that *Xoo* and *Xoc* can both grow in the intercellular spaces between mesophyll cells. But only *Xoc* can break the cell wall junction between mesophyll cells to spread in the extracellular spaces and form streak lesions. 

The cell wall is consisted of cellulose microfibrils embedded in gel-like matrix of hemicellulose (xylan, glucuronoxylan, xyloglucan, arabinoxylan, mixed linkage glucan, or glucomannan), pectic polysaccharides, and minor amounts of structural proteins [27]. The T2SS of *Xoo* can secrete CWDEs such as cellulase, polygalacturonase, xylanase, lipase, and endoglucanase as virulence factors facilitating *Xoo* invading rice [8,16,28]. The T2SS-deficience or the single endoglucanase gene (*eglXoB*) loss of *Xoo* are severely virulence-deficient in rice [28,29]. However, the single mutation of cellulose or lipase gene causes only a partial loss of *Xoo* virulence [16]. The purified celullase, endoglucanase and lipase of *Xoo* can induce rice defense response which are suppressed by the T3SS of *Xoo* [16]. These evidences suggest that the *Xoo* growing in intercellular of mesophyll cells or multiplying in xylem vessels are dependent on the T3SS but independent on the most CWDEs secreted by T2SS [16]. The T2SS-deficient mutant of *Xoc* is also severely virulence-deficient in rice [8]. The T2SS component-XpsD protein and the extracellular protease A (EcpA) secreted by T2SS are tissue-specific virulence factors between *Xoc* strain BLS256 and *Xoo* strains [5,8]. Many genes of cellulase, xylanase and lipase are analyzed in the genome of *Xoc* strains [4,12]. The gene mutants of CWDEs are not screened in *Xoc* strain BLS256 Tn5-mutant library [8] which is possibly because of the CWDEs keeping the housekeeping role in *Xoc* growth [30]. Furthermore, the cell wall of mesophyll cell and the cellulose filter papers were more severely damaged by *Xoc* than that of *Xoo* (Figure 2, Figure 3 and Figure 4). Meanwhile, *Xoc* can break the cell wall of lobes in mesophyll cells to form streak lesions (Figure 1). Therefore, in comparison with *Xoo*, *Xoc* has the stronger cell wall-degradation ability by breaking cell wall linkage of mesophyll cell lobes to facilitate tissue-specific spread in the intercellular spaces between mesophyll cells. The strong cell wall degradation ability of *Xoc*, which may directly determine the tissue-specificity of *Xoc*, should be determined by the CWDEs in T2SS signal pathways. 

A lot of cell wall-related genes of rice are involved in rice-*Xoo*/*Xoc* interactions. Rice major resistance (*MR*) genes *Xa4* and *Xa27* against *Xoo* are associated with the up-regulated expression of cellulose synthase genes and the secondary cell-wall thickening in vascular bundle elements [31,32]. Rice lines with overexpression of *EXPA1*/*5*/*10*, which encoding cell wall-loosening proteins, show increased susceptibility to *Xoo* and *Xoc* [33]. These evidences imply that *Xoo* and *Xoc* may both impair the cell wall mediated resistance in rice. However, no *MR* gene against *Xoc* is identified in rice until now [2], only some cell wall-related genes are associated with rice resistance to *Xoc*. The overexpression of polygalacturonase-inhibiting protein 1/4 (OsPGIP1/4) and small heat shock protein (OsHsp18.0-Cl) genes enhance resistance to bacterial leaf streak in rice [34,35,36]. The polygalacturonase-inhibiting protein functions in inhibiting the activity of enzymes which secreted by bacteria to degrade cell wall component-pectic polysaccharides [34]. The expression of nucleotide-binding site-leucine rich repeats (NB-LRR) maize MR gene *Rxo1*, which mediates rice resistance to *Xoc*, can induce lignin deposition on the inoculation sites [37]. Furthermore, The T3SS defective strain of *Xoc* strain GX01 induces the up-regulated expression of cellulose synthesis enzyme genes and the down-regulated expression of expansin genes encoding cell-wall loosening proteins in *japonica* rice [38]. The expression of cellulose synthase 4/7/9 genes of susceptible rice leaves were induced by *Xoc* to slightly lower levels than control leaves and rice leaves inoculated by *Xoo* (Supplementary Figure S2). The evidences suggest that the cell wall-degradation ability is possibly reinforced by the T3SS effectors of *Xoc*.

## Supplementary Materials

The following are available online at https://www.mdpi.com/2076-0817/9/3/187/s1, Figure S1: No *Xoo* bacterial exudate but many *Xoc* bacterial exudates on leaf of non-inoculation sites, Figure S2: Expression levels of cellulose synthase gene (*CESA*) in IR24 leaves inoculated by PXO99^A^ and RH3, Table S1: PCR primers used for quantitative RT-PCR assays.
