# Supplementary material for: Different Cell Wall-Degradation Ability Leads to Tissue-Specificity between Xanthomonas oryzae pv. oryzae and Xanthomonas oryzae pv. oryzicola
Source: Pathogens. 2020 Mar 4;9(3):187. doi: 10.3390/pathogens9030187 (PMC7157550; doi:10.3390/pathogens9030187)
Supplement: Supplementary file 1 [file pathogens-09-00187-s001.pdf]

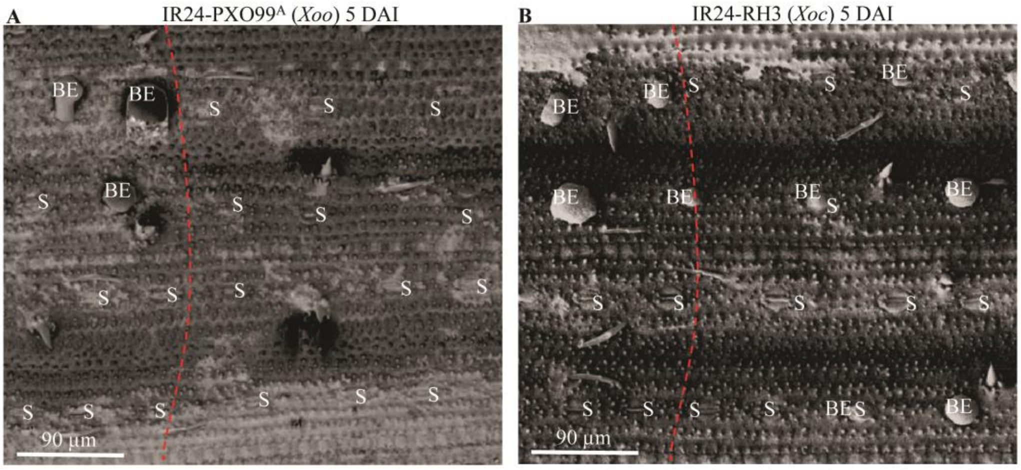

Supplemental Figure 1. No *Xoo* bacterial exudate but many *Xoc* bacterial exudates on leaf of non-inoculation sites. DAI, days after inoculation; BE, bacterial exudates; S, stoma. **(A, B)** The distribution of bacterial exudates on inoculation site (the left of dotted red line) and on non-inoculation site (the right of dotted red line) of IR24 leaves inoculated by *Xoo* strain PXO99^{A} or *Xoc* strain RH3 at 5 DAI.

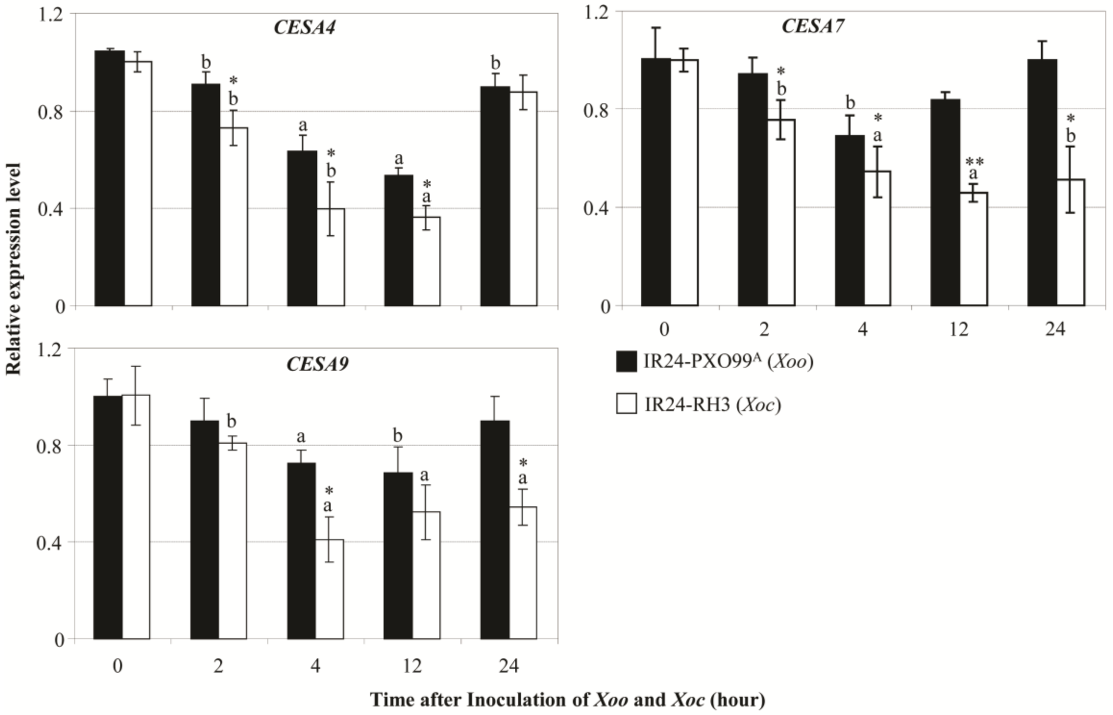

Supplemental Figure 2. Expression levels of cellulose synthase gene (*CESA*) in IR24 leaves inoculated by PXO99^{A} and RH3. IR24 rice plants were inoculated with *Xoo* strain PXO99^{A} and *Xoc* strain RH3 at four-leaf stage. Data are presented as mean (three replicates) ± standard error (SE). Letter "a" and "b" indicate statistically significant differences between 0 h and other time at $P < 0.01$ and $P < 0.05$, respectively in IR24 inoculated with same bacteria. Double asterisks (**$P < 0.01$) and single asterisk (*$P < 0.05$) indicate different levels of statistical significance between IR24 inoculated with PXO99^{A} and IR24 inoculated with RH3 at same time point.

Supplemental Table 1. PCR primers used for quantitative RT-PCR assays

Gene (GenBank accession number or RGAP code) [a]	Forward primer 5′-3′	Reverse primer 5′-3′	Purpose
CESA4 (AK100475)	GTTCGATGGCATTGATCGCA	CCACATAAACCGGACCTTGGA	qRT-PCR
CESA7 (AK072259)	CCGGATGGATGATTCTTGTTG	CCCCCAAAACACTTTTATCCC	qRT-PCR
CESA9 (AK121170)	AGGCCATCCATGTCATCAGCT	TTGAACCCCGTTAGGATGTCC	qRT-PCR
Actin (X15865)	TGTATGCCAGTGGTCGTACCA	CCAGCAAGGTCGAGACGAA	qRT-PCR

[a]RGAP (Rice Genome Annotation Project, http://rice.plantbiology.msu.edu/) locus identifier.
